# Acute severe hepatitis of unknown origin in children in Canada

**DOI:** 10.14745/ccdr.v49i06a02

**Published:** 2023-06-01

**Authors:** Jennifer Macri, Vanessa Morton, Meghan Hamel, Pierre-Luc Trépanier, Marina I Salvadori, Acute Hepatitis Investigation Team

**Affiliations:** 1 Canadian Field Epidemiology Program, Public Health Agency of Canada, Ottawa, ON; 2 Centre for Food-borne, Environmental and Zoonotic Infectious Diseases, Public Health Agency of Canada, Guelph, ON; 3 Centre for Food-borne, Environmental and Zoonotic Infectious Diseases, Public Health Agency of Canada, Ottawa, ON; 4 Ministère de la Santé et des Services sociaux, Montréal, QC; 5 Public Health Agency of Canada, Ottawa, ON; 6 Department of Paediatrics, McGill University, Montréal, QC

**Keywords:** acute severe hepatitis, unknown origin, children, outbreak investigation, administrative databases, baseline incidence

## Abstract

**Background:**

In spring 2022, a series of reports from the United Kingdom and the United States identified an increase in the incidence of acute severe hepatitis in children. The Public Health Agency of Canada (PHAC) collaborated with provincial/territorial health partners to investigate in Canada. Clinical hepatitis, or inflammation of the liver, is not reportable in Canada, so to determine if an increase was occurring above historical levels, the baseline incidence in Canada was estimated. This article estimates the pre-existing baseline incidence of acute severe hepatitis of unknown origin in children in Canada using administrative databases. It further summarizes the outbreak investigation using information from the national case report forms.

**Methods:**

A committee with representatives from PHAC and provincial/territorial health partners was established to investigate current cases in Canada. A national probable case definition and case report form were developed, and intentionally created to be highly sensitive to capture all potential cases for etiological investigations. To estimate a nationally representative baseline incidence, hospitalization data were extracted from the Discharge Abstract Database and was combined with data from Québec from the *Ministère de la Santé et des Services sociaux*.

**Results:**

Twenty-eight probable cases of acute severe hepatitis of unknown origin in children were reported between October 1, 2021, to September 23, 2022, by six provinces: British Columbia=1; Alberta=5; Saskatchewan=1; Manitoba=3; Ontario=14; and Québec=4. The estimated national baseline incidence was an average of 70 cases annually, or 5.8 cases per month.

**Conclusion:**

There was no apparent increase above the estimated historical baseline levels.

## Introduction

On April 5, 2022, the World Health Organization (WHO) was notified of a reported increase in the number of cases of acute severe hepatitis in children in the United Kingdom (UK). These cases of hepatitis were not caused by any known hepatitis virus or other typical causes of hepatitis ((1)). The Public Health Agency of Canada (PHAC) collaborated with provincial/territorial health partners to investigate cases in Canada. Globally, a total of 1,010 probable cases which met the WHO probable case definition have been reported from 35 countries as of July 8, 2022 ((1)).

Hepatitis is an inflammation of the liver ((2)) and can be caused by infectious (usually viral) or non-infectious agents (e.g. alcohol, certain drugs) ((3)). Acute hepatitis ranges widely in severity, from mild to severe, and rarely progresses to liver failure ((3)). Depending on the cause of hepatitis, the progression to liver failure can be acute and rapid, over a few days to weeks, or can occur very slowly ((2)). Acute severe hepatitis in children is a rare condition and the etiology is often unknown ((2)).

Following the initial reports of an increase in cases of acute severe hepatitis of unknown origin, many potential causative agents or associations were hypothesized. Infection with adenovirus 41 was hypothesized due to the detection of the virus in a high proportion of cases in multiple countries: 65% in the UK ((4)) and 45% in the United States ((5)). Adenovirus 41 had not previously been associated with hepatitis in immunocompetent children, which the majority of the cases of acute severe hepatitis were. Additional or abnormal susceptibility, co-infections, environmental exposures or a novel adenovirus variant were also investigated as causative agents ((1,5)). Recent studies have hypothesized association with adeno-associated virus 2 (AAV-2), after identifying this co-infection in cases infected with adenovirus 41 ((5,6)). Adeno-associated virus 2 has not previously been known to be associated with hepatitis ((5–7)) Infection with severe acute respiratory syndrome coronavirus 2 (SARS-CoV-2) was also hypothesized as a cause of this condition due to the ongoing coronavirus disease 2019 (COVID-19) pandemic ((4)). There was no evidence of an association between any SARS-CoV-2 vaccine and acute severe hepatitis in children ((8)).

In Canada, there is no ongoing surveillance for hepatitis cases that are not caused by a hepatitis virus at the national level. Therefore, an estimation of the baseline incidence of acute severe hepatitis of unknown origin in children was needed to ascertain whether an increase in cases was being observed in Canada. The objective of this work was to estimate the baseline number of cases in Canada before 2021 and to investigate the incident cases of acute severe hepatitis of unknown origin in children in Canada as of October 2021.

## Methods

An investigation committee led by PHAC with representatives from each province and territory was established on April 29, 2022. Provinces and territories began active prospective monitoring for cases and conducted a retrospective review up to October 1, 2021 (six months prior to the initial notification of increase of cases by the WHO) to identify any probable cases that met the established case definition. Temporary mandatory reporting or ministry directives were established in each jurisdiction to facilitate national reporting of probable cases. Retrospective chart reviews were conducted by the provinces/territories to identify cases which had occurred between October 1, 2021, but prior to the start of the investigation in April 2022. Any probable cases which met the criteria outlined in the national case definition were included in the national investigation. Surveillance for the purposes of the national investigation was completed on September 23, 2022.

As this condition was not under surveillance prior to this investigation, an estimate of a national baseline incidence was needed to determine if an increase in cases was being detected in Canada. The national investigation was accompanied by a rapid study to estimate the national baseline incidence of the condition prior to October 2021.

### Case definitions

A national probable case definition was adapted from the WHO’s definition in collaboration with provinces and territories.

Probable case definition of acute severe hepatitis of unknown origin in children:

• A person who is 16 years and younger presenting with severe acute hepatitis since October 1, 2021, requiring hospitalization

AND

• With elevated serum transaminase greater than 500 IU/L (AST or ALT)

AND

• Excluding hepatitis caused or attributed to a hepatitis virus (A, B, C, D, E) or a known or expected presentation of a drug or medication; a genetic, congenital, or metabolic condition; an oncologic, vascular, or ischemia-related condition; or an acute worsening of chronic hepatitis

Note: If hepatitis D or E serology results are pending or serology test was not done but other criteria were met, these can be reported as probable cases.

### Epidemiological investigation

A national case report form was developed and shared with provinces and territories. Case report forms were completed with the data available and shared with PHAC. Case report forms included the following information:

• Demographic information (date of birth, sex, ethnicity/race)

• Current case status (status, liver transplantation)

• Illness presentation (symptoms, symptom onset dates, clinical contacts)

• Laboratory results (laboratory markers, infectious diseases, toxicology, medical investigations)

• Medical and health history (COVID-19, previous illnesses, medications, underlying medical conditions, immunosuppression, vaccination)

• Travel history (outside of Canada in the five months prior to diagnosis)

• Other information

### Estimating baseline incidence

To determine whether the incident cases in the active investigation were above the baseline incidence of acute hepatitis of unknown origin in Canada, an estimate of the baseline incidence of the condition was required. Data from the Discharge Abstract Database (DAD) were used to estimate the historical baseline incidence of acute severe hepatitis of unknown origin in children in Canada. The DAD is maintained by the Canadian Institute for Health Information (CIHI) and contains demographic, administrative and clinical information on hospital discharges in Canada. Data from the DAD are regularly shared with PHAC, making this a timely data source to support the active investigation ((9)). Data is submitted to CIHI by acute care facilities, regional health authorities or ministries ((9)). The DAD contains information from all acute inpatient facilities in Canada, with the exception of facilities in Québec ((9)). Data for Québec was extracted from the hospitalization files maintained at the *Ministère de la Santé et des Services sociaux* using the same methodology used to extract data from the DAD. These data were then combined with data from the DAD to estimate a nationally representative baseline.

Inclusion criteria for record extraction from the DAD were established to align as close as possible with the national probable case definition. Records were extracted for individuals 16 years of age and younger, with a primary International Classification of Diseases, Tenth Revision (ICD-10) diagnostic code indicating hepatitis not caused by hepatitis A, B, C or E. The full list of ICD-10 inclusion codes is found in **Table 1**. Extracted cases were then excluded from the analysis if a secondary or contributing diagnostic code indicated a potential known cause for hepatitis, such as a hepatitis virus. The full list of exclusionary ICD-10 codes can be found in **Table 2**. The inclusion and exclusion criteria do not fully align with the national probable case definition because of the different data collection methodologies (passive administrative data versus active case finding).

**Table 1:** ICD-10 codes used for inclusion of cases

ICD-10 code	Description
B17.8	Other specified acute viral hepatitis
B17.9	Acute viral hepatitis, unspecified
B19.0	Unspecified viral hepatitis with hepatic coma
B19.9	Unspecified viral hepatitis without hepatic coma
K72.0	Acute and subacute hepatic failure
K72.9	Hepatic failure, unspecified
K75.2	Nonspecific reactive hepatitis
K75.4	Autoimmune hepatitis
Z94.4	Liver transplant status

Abbreviation: ICD-10, International Classification of Diseases, Tenth Revision

**Table 2:** ICD-10 codes used for exclusion of cases

ICD-10 code	Description
B15.X	Acute hepatitis A
B16.X	Acute hepatitis B
B17.0	Acute delta-(super)infection in chronic hepatitis B
B17.1	Acute hepatitis C
B17.2	Acute hepatitis E
B18.X	Chronic viral hepatitis
K70.1	Alcoholic hepatitis
K73.X	Chronic hepatitis, not elsewhere classified
B25.1	Cytomegaloviral hepatitis
B58.1	Toxoplasma hepatitis
B94.2	Sequelae of viral hepatitis
P35.3	Congenital viral hepatitis
K75.3	Granulomatous hepatitis, not elsewhere classified
Z20.5	Contact with and exposure to viral hepatitis
K71.2	Toxic liver disease with acute hepatitis
Z24.6	Need for immunization against viral hepatitis
K71.3	Toxic liver disease with chronic persistent hepatitis
K71.4	Toxic liver disease with chronic lobular hepatitis
K71.5	Toxic liver disease with chronic active hepatitis
K71.6	Toxic liver disease with hepatitis, not elsewhere classified
B67.8	*Echinococcosis*, unspecified, of liver
K70.X	Alcoholic liver disease
K71.X	Toxic liver disease
K72.1	Chronic hepatic failure
K74.X	Fibrosis and cirrhosis of liver
K75.X	Other inflammatory liver diseases
K76.X	Other diseases of liver
K77	Liver disorders in diseases classified elsewhere
Z52.6	Amoebic liver abscess
C22.9	Malignant neoplasm: liver, unspecified
D13.4	Benign neoplasm: liver
S36.1	Injury of liver or gallbladder
P15.0	Birth injury to liver
Q44.6	Cystic disease of liver
B67.0	*Echinococcosis granulosis* infection of liver
B67.5	*Echinococcosis multilocularis* infection of liver
C18.3	Malignant neoplasm: hepatic flexure
T86.4	Liver transplant failure and rejection
Q44.7	Other congenital malformations of liver
Q26.6	Portal vein-hepatic artery fistula

Abbreviation: ICD-10, International Classification of Diseases, Tenth Revision

Utilizing the national dataset, the average number of cases of acute severe hepatitis of unknown origin was estimated monthly and annually. These cases were then stratified by primary diagnostic codes, which were classified as unspecified hepatitis, hepatic failure or autoimmune hepatitis. The severity of captured cases can be approximated by utilizing hospitalization data the number of severe cases. This approximation aligns with the current case definition for the outbreak investigation, which identified only cases that were hospitalized.

Incidence rates per 100,000 population were determined using the corresponding annual July 1 population estimates from Statistics Canada for people aged 16 years of age and younger ((10)).

A statistical comparison of the estimated national baseline of the condition and the number of cases in the current investigation was not completed. The purpose of estimating the national baseline was to determine if the number of cases in the current investigation exceeded expectation and verify the existence of an outbreak, not to determine the magnitude of the difference between the current investigation and the estimated baseline. Additional statistical testing was not completed due differences between the data collection methodology in the investigation and the methodology used for estimating the national baseline incidence.

## Results

### Baseline incidence

**Figure 1:**
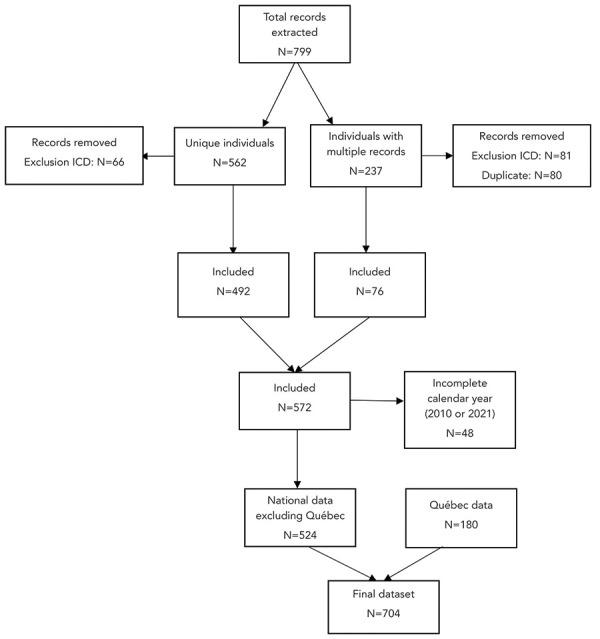
Flow diagram of inclusion, exclusion, deduplication, and appending of data to obtain final dataset Abbreviation: ICD, International Classification of Diseases

The data extracted from the DAD identified a total of 799 records meeting the inclusion criteria. After removing records that met the exclusion criteria, duplicate records and multiple discharges for the same individual, 524 unique individuals were included for analysis. These data were subsequently appended with the data shared from Québec, for a total of 704 cases included in the analysis to estimate the national baseline. A flow chart detailing the inclusion/exclusion, deduplication, and appending of data can be found in **Figure 1**.

**Figure 2:**
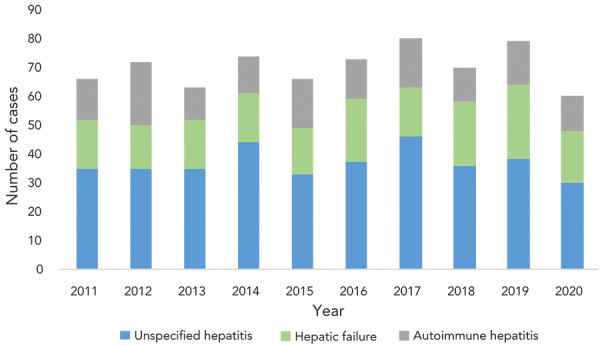
Annual case counts of acute severe hepatitis of unknown origin in children under 16 years of age by diagnostic type in Canada, 2011–2020

An average of 70 (median: 71; range: 60–80) cases of acute severe hepatitis of unknown origin in children were identified per year from 2011 to 2020 in Canada (**Figure 2**). This is an average of approximately 5.83 cases per month. The average annual incidence rate of acute severe hepatitis of unknown origin is 1.14 cases per 100,000 population.

### Epidemiological investigation

**Figure 3:**
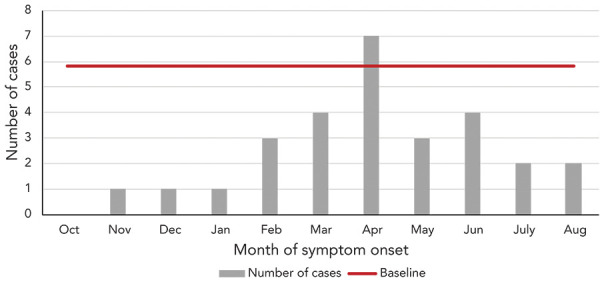
Number of cases of acute severe hepatitis of unknown origin in children by illness onset date, October 1, 2021 to September 23, 2022 (n=28)

A retrospective and prospective investigation for cases since October 1, 2021, was started in May 2022. Provinces and territories used different methods to identify cases. All jurisdictions completed record reviews or requested health care providers to report cases. The investigation of acute severe hepatitis of unknown origin in children was closed on September 23, 2022. In Canada, 28 probable cases of acute severe hepatitis of unknown origin in children were reported to PHAC from October 1, 2021, to September 23, 2022, by six provinces: British Columbia=1; Alberta=5; Saskatchewan=1; Manitoba=3; Ontario=14; and Québec=4. The symptom onset dates for these cases were between November 3, 2021, and August 11, 2022 (**Figure 3**). The highest number of cases (n=7) was identified in April 2022 and exceeded the estimated national baseline of 5.83.

Cases identified were 1–13 years of age with a median age of 5.9 years, and 14 of 28 (50%) cases were five years of age and younger. Among the cases, 15 of 28 (54%) were male. All cases were hospitalized, and seven of 28 (25%) cases were admitted to the intensive care unit/critical care unit.

A total of five of the 26 (19.2%) cases for which testing was completed had a COVID-19 infection confirmed by a polymerase chain reaction (PCR) test during the five months prior to diagnosis (**Table 3**). One additional case reported a respiratory illness during the exposure period but was not tested for COVID-19. The SARS-CoV-2 anti-spike and/or anti-N antibody test results were available for nine cases. Of these nine cases, seven (77.8%) cases were positive and two (22.2%) cases were negative.

**Table 3:** Initial epidemiological data obtained for reported cases of acute severe hepatitis of unknown origin in children 16 years and younger since October 1, 2021

Indicator	Number of cases	Total cases with testing/procedure	Percent (%)
COVID-19, confirmed by PCR	5	26	23.1
Adenovirus (blood or respiratory sample)	6	25	24.0
Liver biopsy	12	27	44.4
Liver transplant	4	27	14.8

Abbreviations: COVID-19, coronavirus disease 2019; PCR, polymerase chain reaction

Adenovirus was detected in blood or respiratory samples of six of the 25 (24%) cases for which testing was completed (Table 3), with one sample typed as B7. The other cases with positive adenovirus samples were not genotyped.

Liver transplant was required for four of the 27 (14.8%) cases (Table 3); however, no significant findings related to the investigation (specifically, a potential cause of the hepatitis) were identified from the explant livers. Liver biopsies were completed for 12 of 27 (44.4%) cases (Table 3) and no significant findings related to the investigation or potential cause of the hepatitis were identified.

## Discussion

Hospitalization data from the DAD was utilized in combination with data from Québec obtained from the *Ministère de la Santé et des Services sociaux* to estimate the number of cases that occur in Canada each year. It was estimated that an average of 70 cases of acute severe hepatitis of unknown origin in children occur each year in Canada, or 5.83 cases per month. On September 23, 2022, at the conclusion of the Canadian outbreak investigation, 28 probable cases (1–7 cases per month) had been reported in Canada from six provinces (an average of 2.3 cases per month). The number of cases exceeded baseline in April by one case, but the number of cases identified was below baseline levels for all other months. Based on these data, and despite the limitations noted below, there does not appear to have been an increase in cases of acute severe hepatitis of unknown origin in children 16 years and younger in Canada during the period of the investigation.

A Canadian national probable case definition was adapted from the WHO’s case definition for the Canadian context. This definition was purposefully designed to capture as many cases as possible, some of which may have had a potential etiology for the hepatitis cases. Conversely, the Canadian case definition added additional specificity compared to the WHO definition, due to the requirement of hospitalization. Less severe cases may not have been captured if not hospitalized. Ensuring the case definition captured all possible severe cases enabled the exploration of possible etiologies, associations or causative agents of the condition. The diagnosis of acute severe hepatitis of unknown origin is not specific and does not imply that all reported cases have the same etiology.

The UK was one of the first countries to identify an increase in acute severe hepatitis in children. Recent articles have suggested potential association due to co-infection with adenovirus and AAV-2 ((6,7)). In the UK investigation, 63.6% of cases tested positive for adenovirus ((5)), whereas in Canada only 24% of cases tested positive for adenovirus. In the UK, most cases (n=214/249, 85.9%) were aged 0–5 years, whereas only 50% of Canadian cases were aged 0–5 years ((7)). In a recent study by Morfopoulou *et al.*, an extensive investigation involving 28 cases and 136 controls in the UK identified high levels of AAV-2 in explanted livers, and in the blood of 10 of 11 (90.1%) non-transplanted cases. The results indicated an association between AAV-2 and acute severe hepatitis in children ((7)). Adeno-associated virus 2 is not routinely tested for in public health laboratories: to our knowledge no Canadian samples were tested for AAV-2. The low number of adenovirus-positive cases and the differences in age demographics, together with the low number of cases in Canada, suggest that the factors leading to an increase in acute severe hepatitis cases in the UK may not have been present in Canada. In the United States, for patients under investigation with available data, approximately 10% identified active SARS-CoV-2 infection and 33% reported a history of SARS-CoV-2 infection ((4)). This is comparable to the Canadian cases identified, as 23.1% of Canadian cases had a COVID-19 infection confirmed by PCR in the five months prior to the diagnosis. Therefore, it is likely that the number of cases reported in Canada reflects the baseline level of acute severe hepatitis of unknown origin in children routinely observed in Canada.

### Strengths and limitations

This is the first identification of a baseline incidence of acute severe hepatitis of unknown origin in children in Canada. The DAD captures administrative, clinical and demographic data from all acute care facilities or their respective health or regional ministry or department of health, with the exception of Québec ((9)). With the addition of the data obtained from the *Ministère de la Santé et des Services sociaux* in Québec, these data provide a nationally representative estimate of the incidence, which was not established prior to this investigation.

A limitation of this approach is the methodological differences in case classifications between: 1) estimating the national baseline incidence using passive administrative data and 2) active case finding during the investigation. Without completing a comprehensive chart review of all cases included in the baseline analysis, which was not possible due to the time constraints of an active investigation, there is potential for misclassification and reduced comparability between the cases captured in the current outbreak and in the baseline estimate. Additionally, without completing a case-by-case comprehensive medical chart review for each case included in the baseline estimate, there is the potential for misclassification and reduced comparability between the cases captured in the current outbreak and the estimate of the baseline incidence. As active case finding was used during the current investigation, it is unlikely to have underrepresented the incidence during that period. Finally, ICD-10 diagnostic coding was used to estimate the baseline incidence but not in the investigation’s case definition; thus, the cases captured in the baseline estimate may not have completely aligned with those captured in this investigation. This may have resulted in an over or under-estimation of the baseline incidence compared to the condition under investigation. However, for the purposes of this investigation, where an estimate of the national baseline incidence was rapidly required to determine if the condition being investigation was exceeding the estimated baseline number of cases, the national estimate from the DAD provided sufficient evidence to be used in conjunction with other sources of evidence to support the investigation.

For the investigation, conducting retrospective review of records for cases since October 1, 2021, was difficult to complete due to resource-intensive chart reviews, transfers of patients between facilities and jurisdictions, and different methodologies used between jurisdictions. This limits the comparability of estimates between provinces and territories, and results in potential under or overestimation of cases based on the methodology used.

## Conclusion

This federal/provincial/territorial joint investigation was able to identify cases of acute hepatitis of unknown origin in Canada. The analysis of hospital records provided an estimate of the baseline incidence of acute severe hepatitis of unknown origin in children in Canada on this information, and an increase above the expected historic baseline for this condition was not observed. More research is required internationally to identify the possible cause of the increase of acute severe hepatitis in children observed in certain regions and to fully elucidate any possible links to adenovirus, AAV-2 or other potential causation.
